# The complete chloroplast genome sequence of *Dryobalanops aromatica*

**DOI:** 10.1080/23802359.2021.1914223

**Published:** 2021-05-21

**Authors:** Jihua Wang, Weizhong Huang, Shike Cai, Junliang Zhao

**Affiliations:** a Key Laboratory of Crops Genetic Improvement of Guangdong, Crops Research Institute, Guangdong Academy of Agricultural Sciences, Guangzhou, China; b Guangdong Luofushan Sinopharm Co., Ltd, Huizhou, China; c Rice Research Institute & Guangdong Key Laboratory of New Technology in Rice Breeding, Guangdong Academy of Agricultural Sciences, Guangzhou, China

**Keywords:** *Dryobalanops aromatica*, Euphorbia, chloroplast genome, medical plant

## Abstract

*Dryobalanops aromatica* is a new species in the family of Lauraceae with high content of D-borneol, which is an important raw material of premium spices and medicine widely used in China. The genome and the molecular phylogenetic relation of this novel species had not been analyzed before. In this study, we present the complete sequence of chloroplast genome of *D. aromatic,* as well as its genome annotation. The complete chloroplast sequences in length were 152,696 bp, with two single-copy regions 93,610 bp and 18,902 bp in length, which were separated by two inverted repeat regions with 20,092 bp in length. In total, 128 genes were predicted with GC content at 39.16%. Phylogenetic analysis showed that *D. aromatica* is closest to *Gossypium sturtianum* in Lauraceae. The sequence and annotation of the chloroplast genome of *Dryobalanops aromatic* will be useful for further studies on the taxonomy in Lauraceae.

*Dryobalanops aromatica* is belonging to the family of Lauraceae, which is an important Chinese herb medical plants and only distribute in Pingyuan city, China. It is a special species in Lauraceae with a very high content of D-borneol. It is an important raw material of premium spices and medicine used widely, which has the functions as heart, brain and other organs protecting, central nervous system regulating and promoting other drugs absorption (Chen et al. 2011; Fu et al. 2020). Though it is very important to category analysis on the Lauraceae, the sequence information of D. aromatica genomics has not been reported. On the other hand, in medical plants, it is important to develop series of genus-specific DNA barcode for identity-based on the full chloroplast sequences. Next-generation sequencing (NGS) technologies provide the high-throughput and efficient methods for chloroplast genome research(Wang et al. 2018).In this study, we reported the complete chloroplast sequence and the annotation of *D. aromatica*, as well as the phylogenetic analysis base on the assembled sequence by NGS. The results of the present study will be useful for evolutionary and molecular biological research of *D. aromatica*.

The leaves of *D. aromatica* were collected at the Shizheng (24°32'21.27''N, 115°52'41.33''E), Meizhou city, Guangdong province, China. The young leaves were collected and frozen by liquid nitrogen and stored at −80 °C refrigerator at the Key Laboratory for Crops Genetic Improvement of Guangdong in Guangdong Academy of Agricultural Sciences(specimen code Mps2020). The genomic DNA of *D. aromatica* was extracted by plant genomic DNA kit (Omega) from the leaves and sequenced by the Novaseq platform (Illumina, San Diego, CA) following as the recommended manufacturer. After quality assessment and filtering, the 5 G clean data was used to assemble the sequence of the chloroplast genome by the GetOrganelle v1.6.2e (Jin et al. 2019) and annotated with the Geseq (Tillich et al. 2017). Then, the sequence were submitted to GenBank with the accession number MW373534.

The complete chloroplast genome of *D. aromatica* assembled in this study is 152,696 bp in length. It has two single-copy regions of 93,610 bp and 18,902 bp, which are separated by the same two inverted repeat (IR) region with 20,092 bp. In total, 128 genes were predicted, including 84 protein-coding genes, 36 tRNA genes, and 8 rRNA genes. The GC content of the chloroplast genome is 39.16%.

To compare the relationship between *D. aromatica* and other 13 genera in the other Cinnamomum, a phylogenetic tree was constructed with the complete chloroplast sequence of these species (*Neobalanocarpus heimii*, *Hopea dryobalanoides*, *Hopea reticulata*, *Hopea hainanensis*, *Shorea pachyphylla*, *Shorea pachyphylla*, *Parashorea macrophylla*, *Dipterocarpus turbinatus*, *Vatica odorata*, *Vatica mangachapoi*, *Vatica guangxiensis*, *Gossypium sturtianum* and *Bixa orellana*), which were downloaded from the NCBI (www.ncbi.nlm.nih.gov), using MAFFT v7.407 (Katoh and Standley 2013). The maximum likelihood tree was constructed with RaxML v8.2.12 with 1000 bootstraps(Stamatakis 2014) (Figure 1). The results showed that *D. aromatica* was closest to *Gossypium sturtianum*.

**Figure 1.**
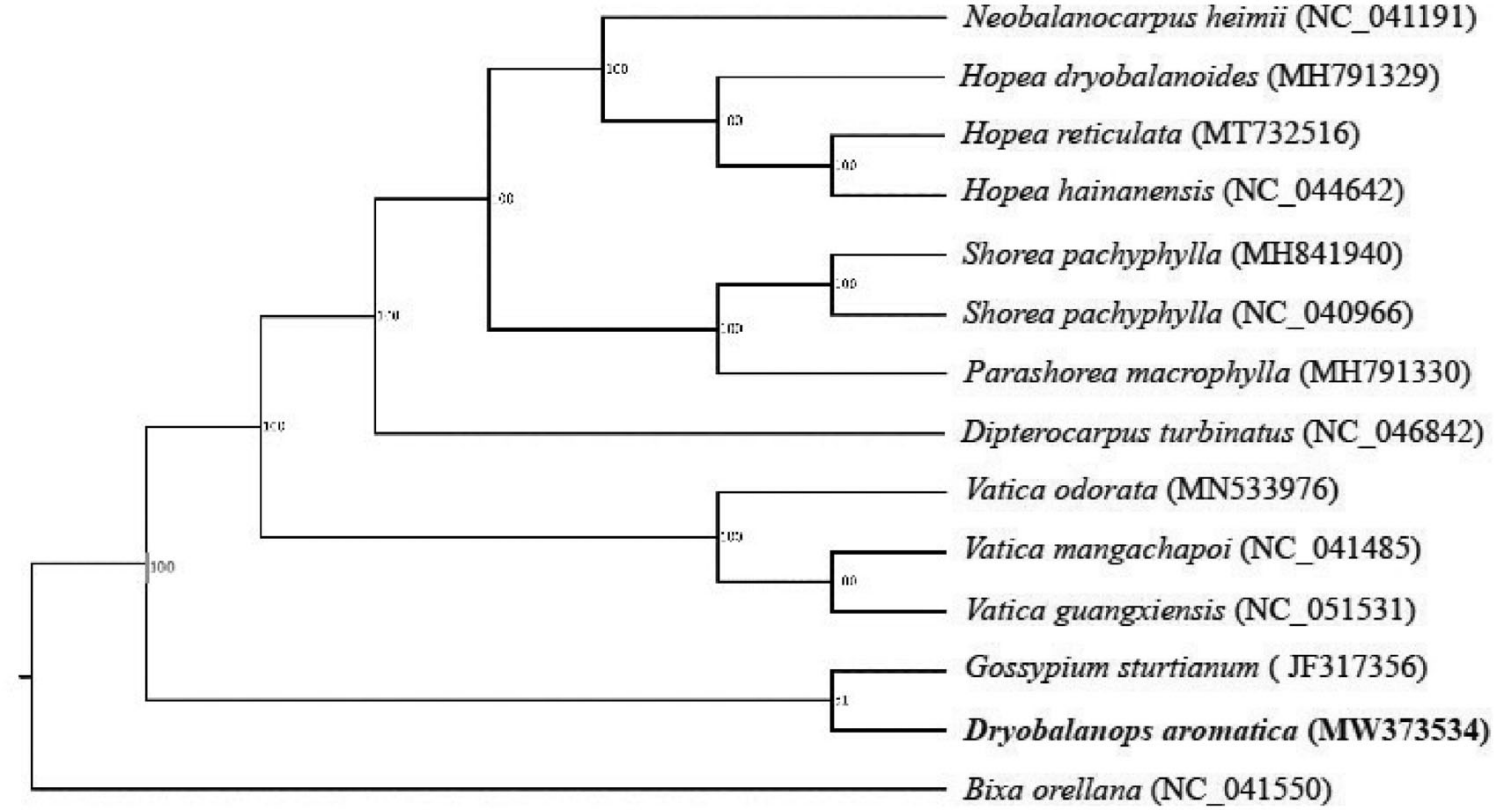
The phylogenetic tree of the *D. aromatica* and similar species was constructed based on 14 full chloroplast sequences.

## Data Availability

The genome sequence data that support the findings of this study are openly available in GenBank of NCBI at (https://www.ncbi.nlm.nih.gov/) under the accession no. MW373534. The associated SRA numbers are PRJNA689327.

## References

[CIT0001] Chen L, Su JY, Li L, Li B, Li W. 2011. A new source of natural D-borneol and its characteristic. J Med Plants Res. 5:3440–3447.

[CIT0002] Fu M, Lu Z, Ma X. 2020. Enhanced extraction efficiency of natural D-borneol from Mei Pian tree leaves pretreated with deep eutectic solvents. Food Sci Nutr. 8(7):3806–3813. 32724642 10.1002/fsn3.1671 PMC7382189

[CIT0003] Jin JJ, Yu WB, Yang JB, Song Y, Yi TS, Li DZ. 2019. GetOrganelle: a fast and versatile toolkit for accurate de novo assembly of organelle genomes. bioRxiv, 256479. 10.1186/s13059-020-02154-5 PMC7488116 32912315

[CIT0004] Katoh K, Standley DM. 2013. MAFFT multiple sequence alignment software version 7: improvements in performance and usability. Mol Biol Evol. 30(4):772–780. 23329690 10.1093/molbev/mst010 PMC3603318

[CIT0005] Stamatakis A. 2014. RAxML version 8: a tool for phylogenetic analysis and post-analysis of large phylogenies. Bioinformatics. 30(9):1312–1313. 24451623 10.1093/bioinformatics/btu033 PMC3998144

[CIT0006] Tillich M, Lehwark P, Pellizzer T, Ulbricht-Jones ES, Fischer A, Bock R, Greiner S. 2017. GeSeq - versatile and accurate annotation of organelle genomes. Nucleic Acids Res. 45(W1):W6–W11. 28486635 10.1093/nar/gkx391 PMC5570176

[CIT0007] Wang X, Cheng F, Rohlsen D, Bi C, Wang C, Xu Y, Wei S, Ye Q, Yin T, Ye N. 2018. Organellar genome assembly methods and comparative analysis of horticultural plants. Hortic Res. 5:3. 29423233 10.1038/s41438-017-0002-1 PMC5798811

